# Apparent Diffusion Coefficient on MRI and Pathological T Stage in Laryngeal Squamous Cell Carcinoma

**DOI:** 10.3390/jimaging12100485

**Published:** 2026-10-06

**Authors:** Pasquale Frisina, Filippo Valentini, Marco De Vincentis, Alessandro Corsi, Anna Borghetti, Alessia Matrigiani, Antonio Iacuzio, Paolo Izzo, Francesca De Felice, Valeria Panebianco, Iacopo Carbone, Daniela Messineo

**Affiliations:** 1 Department of Radiological, Oncological and Pathological Sciences, Sapienza University of Rome, 00185 Rome, Italy; 2 Department of Sense Organs, Sapienza University of Rome, 00185 Rome, Italy; 3 Department of Molecular Medicine, Sapienza University of Rome, 00161 Rome, Italy; 4 “Pietro Valdoni” Department of Surgery, Policlinico “Umberto I”, Sapienza University of Rome, 00161 Rome, Italy

**Keywords:** laryngeal squamous cell carcinoma, apparent diffusion coefficient, diffusion-weighted imaging, magnetic resonance imaging, pathological T stage, preoperative staging, head and neck cancer

## Abstract

Preoperative differentiation between pathological T3 (pT3) disease, representing locally advanced tumor confined to the larynx, and pathological T4 (pT4) disease, characterized by extension through the laryngeal cartilage and/or beyond the larynx, remains challenging in laryngeal squamous cell carcinoma (LSCC). This single-center retrospective study investigated the association between primary-tumor apparent diffusion coefficient (ADC) and pathological T stage and explored whether ADC could complement MRI-based morphological T classification. Twenty-eight patients with surgically confirmed, moderately differentiated LSCC were included (13 pT3 and 15 pT4). Mean ADC values were compared using the Mann–Whitney U test, and discrimination of pT4 disease was evaluated using receiver operating characteristic (ROC) analysis. Leave-one-out cross-validation was performed for internal validation of the ADC threshold and exploratory combined MRI–ADC rule. Mean ADC was significantly lower in pT4 than in pT3 tumors (760 ± 209 versus 1150 ± 188 × 10^−6^ mm^2^/s; *p* < 0.001). ADC achieved an area under the ROC curve of 0.913 (95% confidence interval, 0.769–1.000). The apparent combined MRI–ADC rule yielded a sensitivity of 93.3%, specificity of 100%, and overall accuracy of 96.4%. Under leave-one-out cross-validation, the corresponding estimates were 93.3%, 92.3%, and 92.9%, respectively. No statistically significant association between primary-tumor ADC and pathological nodal status was detected. Primary-tumor ADC was associated with pathological T stage and showed potential complementary value to morphological MRI; however, the threshold and combined rule remain hypothesis-generating and require independent external validation.

## 1. Introduction

Laryngeal squamous cell carcinoma (LSCC) is a major head and neck malignancy with substantial implications for survival, functional preservation, and quality of life [1]. Accurate preoperative assessment of local tumor extent is essential for treatment planning, particularly in locally advanced disease, in which the distinction between T3 and T4 tumors may influence the choice between organ-preservation strategies and more radical surgical approaches.

Cross-sectional imaging plays a central role in the preoperative staging of LSCC. Computed tomography (CT) provides high spatial resolution and is particularly useful for assessing laryngeal cartilage abnormalities; however, its sensitivity for detecting cartilage penetration and subtle extralaryngeal extension remains limited. Moreover, extralaryngeal spread may occur without overt cartilage penetration, further complicating morphological staging. Magnetic resonance imaging (MRI), owing to its superior soft-tissue contrast, may improve the evaluation of deep laryngeal spaces, cartilage involvement, and extralaryngeal extension. Nevertheless, inflammatory and reactive changes may mimic tumor infiltration on MRI and reduce its specificity [2].

Diffusion-weighted imaging (DWI) provides functional information complementary to morphological MRI [3,4]. The apparent diffusion coefficient (ADC) quantitatively reflects water diffusion within tissues and is influenced by tumor microstructure. In malignant lesions, lower ADC values are generally associated with increased cellularity and reduced extracellular space. Histopathological correlation studies in laryngeal and hypopharyngeal squamous cell carcinoma have demonstrated inverse relationships between ADC and cellular density, nuclear area, and nucleus-to-cytoplasm ratio, together with a positive association with stromal content [5]. These observations provide a biological rationale for investigating whether diffusion-derived microstructural information may be associated with local disease extent [6,7].

Previous studies in head and neck cancer have investigated ADC for lesion characterization, lymph node assessment, and the prediction or early evaluation of treatment response [8,9,10,11]. However, its potential contribution to preoperative MRI-based morphological T classification of laryngeal carcinoma remains less clearly established. In particular, it is uncertain whether quantitative diffusion information can complement morphological MRI in differentiating T3 from T4 disease and identifying tumors at risk of radiological understaging.

Throughout this study, the prefix “p” denotes pathological classification based on examination of the surgical specimen. Accordingly, pT3 and pT4 refer to pathological primary-tumor categories, whereas T categories without the “p” prefix refer to MRI-based classification. The designation pT4a indicates moderately advanced local disease with extension through the outer cortex of the thyroid cartilage and/or into tissues beyond the larynx. Pathological nodal status was categorized as pN0, indicating the absence of pathologically confirmed regional nodal involvement, or pN+, indicating the presence of one or more pathologically confirmed regional lymph-node metastases. Imaging-based N0 indicates no regional nodal involvement identified on imaging.

Therefore, the primary aim of this study was to investigate the association between primary-tumor ADC and pathological T stage (pT3 versus pT4) in a histologically homogeneous cohort of moderately differentiated LSCC. We additionally explored the ability of ADC to discriminate between pT3 and pT4 disease and the potential complementary value of integrating ADC with MRI-based morphological T classification. The association between primary-tumor ADC and pathological nodal status was evaluated as a secondary exploratory objective.

## 2. Materials and Methods

### 2.1. Study Design and Patient Population

This single-center retrospective observational study screened patients who underwent subtotal or total laryngectomy for laryngeal squamous cell carcinoma (LSCC) at Policlinico Umberto I, Rome, between 2018 and 2025. The study was intentionally restricted to surgically treated patients with moderately differentiated (G2) pT3 or pT4 LSCC to reduce heterogeneity related to histological type, grade, and pathological stage. This restriction resulted in a selected disease spectrum and was not intended to represent the entire population undergoing preoperative imaging evaluation for LSCC. All procedures involving human participants complied with the ethical standards of the 1964 Declaration of Helsinki and its subsequent amendments [12]. The use of anonymized clinical and imaging data was authorized by the Department of Radiological, Oncological and Pathological Sciences, as documented in the Department Council meeting of 16 December 2025. All data were anonymized before analysis. 

The study was reported in accordance with the Strengthening the Reporting of Observational Studies in Epidemiology (STROBE) recommendations, and for its diagnostic-accuracy component, the Standards for Reporting Diagnostic Accuracy Studies (STARD 2015) statement [13,14]. The completed STARD 2015 checklist is provided as Supplementary File S1.

Patients were excluded for any of the following reasons: (1) histological type other than squamous cell carcinoma or histological grade other than G2; (2) pathological T category other than pT3 or pT4; (3) unavailable or technically inadequate DWI or ADC maps; (4) unavailable definitive surgical histopathology; or (5) salvage laryngectomy after previous radiotherapy or chemoradiotherapy. A total of 68 potentially eligible patients were screened. Thirty-seven were excluded because the histological diagnosis was unavailable (*n* = 22), the tumor was not a squamous cell carcinoma (*n* = 2), the pathological T category was other than pT3 or pT4 (*n* = 2), or the pathological T category was unavailable (n = 11).The remaining 31 patients had surgically confirmed pT3–pT4 LSCC. Of these, two were excluded because of poorly differentiated G3 histology and one because the ADC images were technically inadequate for reliable quantitative measurement. The final study population therefore comprised 28 patients with moderately differentiated G2 LSCC. Pathological T and N categories were obtained from the definitive surgical pathology reports and assigned according to the eighth edition of the American Joint Committee on Cancer (AJCC) staging system. Definitive pathological staging was based on the routine comprehensive examination of the surgical specimen. For pT4a tumors, the definitive pathology reports were additionally reviewed to characterize the anatomical basis of the pathological T classification, with particular attention to thyroid cartilage invasion and extension beyond the laryngeal framework into adjacent soft tissues or structures.

### 2.2. MRI Acquisition

MRI examinations were performed using a 3-T GE Discovery MR750 system (GE Medical Systems, Waukesha, WI, USA) with a dedicated head-and-neck coil. The imaging protocol included sagittal, coronal, and axial T2-weighted fast spin-echo (FSE) sequences, together with axial fat-suppressed T2-weighted FSE and T2-weighted PROPELLER acquisitions. T1-weighted imaging included axial pre- and post-contrast acquisitions, an axial fat-suppressed post-contrast sequence, and delayed fat-suppressed three-dimensional T1-weighted LAVA FLEX acquisitions. Dynamic contrast-enhanced imaging was performed using an axial DISCO sequence. Diffusion-weighted imaging (DWI) was acquired in the axial plane using b-values of 50, 500, and 1000 s/mm^2^. Apparent diffusion coefficient (ADC) maps were subsequently generated using the manufacturer-provided reconstruction software.

For the axial T2-weighted FSE sequence, the acquisition parameters were as follows: TR/TE, 9686/106.66 ms; field of view, 352 mm; matrix, 512 × 512; in-plane voxel size, 0.6875 × 0.6875 mm; slice thickness/spacing, 3/3.5 mm; and flip angle, 111°. For DWI, the corresponding parameters were as follows: TR/TE, 4118/54.4 ms; field of view, 280 mm; matrix, 256 × 256; in-plane voxel size, 1.09375 × 1.09375 mm; slice thickness/spacing, 3/3.5 mm; and flip angle, 90°.

Post-contrast sequences were acquired following the intravenous administration of gadoterate meglumine (Dotarem, Guerbet, Roissy Charles de Gaulle Cedex, France) or gadoteridol (ProHance, Bracco, Milan, Italy). No substantive modifications to the MRI acquisition protocol occurred during the study period.

### 2.3. Radiological Staging and ADC Measurement

MRI-based morphological T classification was retrospectively assessed by two radiologists with 25 and 5 years of experience in head-and-neck imaging, respectively. The examinations were initially reviewed independently, and disagreements were resolved by consensus. The assessment was based exclusively on MRI, without access to laryngoscopic findings, positron emission tomography/computed tomography (PET/CT), other imaging examinations, postoperative histopathological findings, or the original MRI reports.

Tumors were classified according to the eighth edition of the American Joint Committee on Cancer (AJCC) staging system, using anatomical criteria assessable on MRI. For glottic tumors, MRI T3 classification was assigned when the tumor remained confined to the larynx but involved the paraglottic space and/or the inner cortex of the thyroid cartilage. For supraglottic tumors, involvement of the pre-epiglottic space or postcricoid area was also considered a T3 criterion. Vocal-cord fixation is included among the AJCC T3 criteria; however, because the readers did not have access to laryngoscopic findings, vocal-cord mobility or fixation was not independently assessed during the retrospective MRI review.

MRI T4a classification was assigned when the tumor extended through the outer cortex of the thyroid cartilage and/or invaded tissues beyond the larynx, including the trachea, cricoid cartilage, cervical soft tissues, deep extrinsic muscles of the tongue, strap muscles, thyroid gland, or esophagus, as applicable to the primary tumor subsite. MRI T4b classification was defined by invasion of the prevertebral space, encasement of the carotid artery, or invasion of mediastinal structures [15]. At the T3–T4 boundary, thyroid cartilage penetration represented the principal anatomical feature assessed by MRI-based morphological T classification. Extralaryngeal extension was considered when present according to AJCC criteria but was not analyzed as a separate study endpoint.

Cartilage involvement was evaluated on T2-weighted and contrast-enhanced T1-weighted images by comparing areas of abnormal cartilage signal with the adjacent tumor and assessing cartilage integrity. Cartilage signal intensity similar to that of the adjacent tumor was considered suggestive of neoplastic invasion, whereas signal intensity greater than that of the tumor was considered more suggestive of peritumoral inflammatory change, in accordance with previously described MRI criteria [16]. Paraglottic- and pre-epiglottic-space involvement was defined by replacement of the normal fat signal by contiguous tumor tissue. Extralaryngeal extension was defined as contiguous tumor tissue extending beyond the external laryngeal framework into adjacent soft tissues. ADC measurements were not used for MRI-based morphological T classification.

The original MRI reports had not been authored by either retrospective reader and were accessed only after the independent assessments had been completed and all disagreements had been resolved by consensus. The final retrospective consensus classification was subsequently compared with the T stage reported in the original preoperative MRI report. The retrospective consensus classification was used as the MRI-based morphological T classification in all subsequent diagnostic-performance analyses. Image review and quantitative ADC measurements were performed using Agfa Enterprise Imaging (Agfa Health Care N.V., Mortsel, Belgium) and its integrated region-of-interest measurement tool.

For quantitative diffusion analysis, the primary tumor was localized by correlating anatomical T1- and T2-weighted images with DWI and the corresponding ADC maps. Three manually defined regions of interest (ROIs) were placed within solid tumor tissue on three separate axial sections to sample spatially distinct portions of the lesion. ROI placement on the ADC maps was guided by side-by-side correlation with the corresponding T1-weighted, T2-weighted, diffusion-weighted, and contrast-enhanced images. Each ROI was positioned entirely within the visible tumor boundaries. Visually identifiable necrotic components, susceptibility or motion artifacts, peripheral tumor margins, and adjacent non-neoplastic tissues were excluded to minimize partial-volume contamination. Because the three ROIs were placed on different axial sections, they could not be displayed simultaneously on a single ADC-map image.

The three ROIs had a combined area of approximately 200 mm^2^. No ADC measurements were obtained from non-neoplastic reference tissues; their exclusion referred exclusively to the avoidance of partial-volume contamination during tumor ROI placement. This approach was selected to sample spatially distinct solid portions of the tumor while allowing reliable ROI placement in relatively small or irregularly shaped laryngeal lesions.

ADC values were expressed as ×10^−6^ mm^2^/s. The three ROI measurements were averaged to derive a single tumor ADC_mean value for each examination. Both radiologists independently performed ADC measurements while blinded to pathological stage and to each other’s measurements. Measurements obtained during the first assessment by the first radiologist were used for the primary statistical analyses, whereas those obtained by the second radiologist were used to assess interobserver reliability. To assess intraobserver reliability, the first radiologist repeated the measurements after a four-week interval while blinded to the initial measurements.

Intra- and interobserver reliability of tumor ADC_mean measurements was assessed using two-way mixed-effects, absolute-agreement, single-measure intraclass correlation coefficients (ICCs) with corresponding 95% confidence intervals (CIs). ICC values < 0.50 were interpreted as indicating poor reliability, values from 0.50 to <0.75 as moderate reliability, values from 0.75 to 0.90 as good reliability, and values > 0.90 as excellent reliability.

### 2.4. Statistical Analysis

Continuous variables were summarized using the mean and standard deviation (SD), together with the median and range where appropriate. Patient age was calculated at the date of the preoperative MRI examination. The MRI-to-surgery interval was calculated as the number of days between the preoperative MRI examination and the surgical procedure. Categorical variables were summarized as counts and percentages. Demographic and clinicopathological characteristics were compared between the pT3 and pT4 groups. Age was compared using the Mann–Whitney U test, whereas sex, primary tumor site, and pathological nodal status were compared using Fisher’s exact test. For the primary ADC analysis, patients were stratified according to pathological T category into pT3 and pT4 groups. For the secondary ADC analysis of nodal status, patients were categorized as pN0 or pN+.

Between-group differences in primary-tumor ADC were assessed using the Mann–Whitney U test. The magnitude of each difference was additionally estimated using the Hodges–Lehmann estimator, calculated as pT3 minus pT4 for the primary analysis and pN0 minus pN+ for the secondary analysis.

The ability of primary-tumor ADC to discriminate pT4 from pT3 tumors was evaluated using receiver operating characteristic (ROC) curve analysis. The area under the ROC curve (AUC) was calculated with a percentile-based 95% confidence interval (CI) obtained from 5000 nonparametric bootstrap resamples. An exploratory optimal ADC threshold was internally derived using the Youden index. Sensitivity and specificity at the identified threshold were subsequently calculated.

Using the internally derived Youden threshold, an exploratory ADC threshold-based categorization was defined, with tumors showing ADC values at or below the threshold categorized as pT4. Confusion matrices were generated for three approaches: MRI-based morphological T classification, ADC threshold-based categorization, and an exploratory combined MRI–ADC rule. Under the combined rule, tumors were categorized as pT4 when either MRI-based morphological T classification assessment indicated T4 disease or ADC was at or below the internally derived threshold. Because this OR combination is inherently designed to increase sensitivity, its performance was evaluated descriptively and was not interpreted as demonstrating superiority or incremental diagnostic benefit. Pathological T stage was used as the reference standard. Sensitivity, specificity, positive predictive value (PPV), negative predictive value (NPV), overall accuracy, and Cohen’s κ were calculated for each approach.

Because the ADC threshold was initially derived and evaluated in the same cohort, leave-one-out cross-validation (LOOCV) was performed for internal validation. At each iteration, one patient was held out, and the optimal ADC threshold was recalculated using the Youden index in the remaining 27 patients. The resulting threshold was then applied to the held-out patient. This procedure was repeated for all 28 patients, generating one cross-validated ADC threshold-based prediction for each patient. The exploratory combined MRI–ADC rule was evaluated using the same procedure: the held-out patient was categorized as pT4 when either MRI-based morphological T classification indicated T4 disease or the patient’s ADC value was at or below the threshold derived exclusively from the corresponding 27-patient training set. The full-cohort threshold and its associated performance measures were retained as apparent in-sample estimates and reported separately from the LOOCV estimates.

The association between ADC and pathological T category was further quantified using univariable logistic regression, with pT4 as the outcome. This analysis was intended to estimate the association between continuous ADC and the odds of pT4 disease and was not developed or evaluated as a predictive classification model. For clinical interpretability, ADC was rescaled so that regression coefficients and odds ratios (ORs) represented a 100 × 10^−6^ mm^2^/s increase, with corresponding 95% CIs and *p*-values.

Exact 95% CIs for apparent sensitivity, specificity, PPV, NPV, and overall accuracy were calculated using the Clopper–Pearson method. For LOOCV performance estimates, Clopper–Pearson intervals were calculated from the aggregated held-out predictions and are reported as descriptive binomial intervals. Because predictions across LOOCV iterations are not statistically independent owing to the overlap among training sets, these intervals do not account for the dependence introduced by the cross-validation procedure and should not be interpreted as formal inferential confidence intervals for out-of-sample performance. The exact McNemar test was used to assess asymmetry between MRI understaging and overstaging relative to pathological T stage. Apparent and cross-validated performance estimates were interpreted as exploratory and compared descriptively without formal testing of superiority.

The secondary relationship between primary-tumor ADC and pathological nodal status was explored using the Mann–Whitney U test, Hodges–Lehmann estimator, ROC analysis, and univariable logistic regression, with pN+ as the outcome. Given the exploratory nature of this secondary analysis, no ADC threshold for pathological nodal status was derived.

Interobserver agreement for the initial MRI-based morphological T classification, before consensus, was assessed using Cohen’s κ with a percentile-based 95% CI obtained from 5000 nonparametric bootstrap resamples. The proportion of observed agreement and the number of initially discordant classifications were also reported. Interobserver agreement was interpreted as a measure of reader reproducibility, whereas diagnostic accuracy was calculated separately using the final consensus MRI-based morphological classification.

All statistical analyses were performed using Python (version 3.12) with NumPy, pandas, SciPy, scikit-learn, and statsmodels. All tests were two-sided, and *p* < 0.05 was considered statistically significant. Given the exploratory nature of the study and its limited sample size, no adjustment for multiple comparisons was performed.

## 3. Results

### 3.1. Study Population

**Figure 1:**
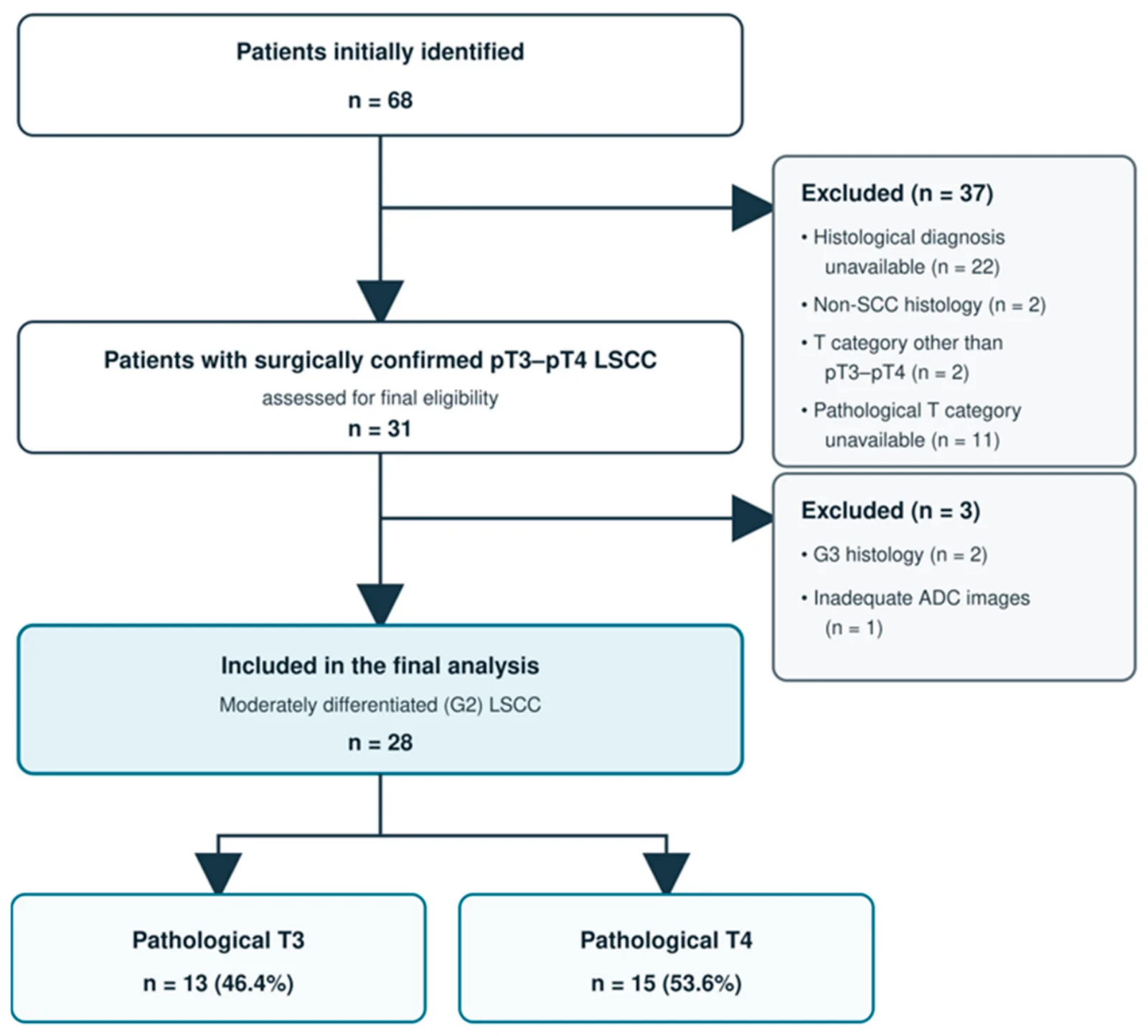
Patient-selection flow diagram. A total of 68 patients were initially identified. Thirty-seven were excluded because the histological diagnosis was unavailable (n = 22), the histological type was other than squamous cell carcinoma (n = 2), the pathological T category was other than pT3–pT4 (n = 2), or the pathological T category was unavailable (n = 11). Of the remaining 31 patients with surgically confirmed pT3–pT4 laryngeal squamous cell carcinoma, two were excluded because of G3 histology and one because the ADC images were technically inadequate for reliable quantitative analysis. The final cohort consisted of 28 patients with moderately differentiated G2 tumors, including 13 pT3 and 15 pT4 cases. ADC, apparent diffusion coefficient; LSCC, laryngeal squamous cell carcinoma; SCC, squamous cell carcinoma.

A total of 68 patients were initially identified. Thirty-seven were excluded because the histological diagnosis was unavailable (n = 22), histology was other than squamous cell carcinoma (n = 2), the pathological T category was other than pT3–pT4 (n = 2), or pathological T classification was unavailable (n = 11). Of the remaining 31 patients with surgically confirmed pT3–pT4 LSCC, two were excluded because of G3 histology and one because the ADC images were technically inadequate for reliable quantitative analysis. The final cohort consisted of 28 patients with moderately differentiated G2 LSCC and analyzable preoperative DWI/ADC data (Figure 1).

The cohort comprised 24 men (85.7%) and 4 women (14.3%), with a mean age at preoperative MRI of 63.5 ± 8.2 years (range, 44–82 years). The median interval between preoperative MRI and surgery was 26.5 days (range, 2–79 days). The primary tumor was located in the glottis in 16 patients (57.1%) and in the supraglottis in 12 (42.9%). According to pathological T stage, 13 patients (46.4%) had pT3 disease and 15 (53.6%) had pT4 disease. All 15 pT4 tumors were classified as pT4a; no pT4b tumors were included. Review of the definitive pathology reports showed heterogeneous patterns of locally advanced disease underlying the pT4a classification. These included thyroid cartilage invasion, in some cases extending through the full thickness of the cartilage, and extension beyond the laryngeal framework into adjacent soft tissues or structures. Cartilage invasion and extralaryngeal extension could coexist within the same tumor, with the latter ranging from focal involvement of the prelaryngeal soft tissues to involvement of other adjacent structures. Regarding pathological nodal status, 17 patients (60.7%) were classified as pN0 and 11 (39.3%) as pN+. Demographic and clinicopathological characteristics stratified according to pathological T stage are summarized in Table 1. Age, sex, and primary tumor site did not differ significantly between the pT3 and pT4 groups. Pathological nodal involvement was more frequent in the pT4 group than in the pT3 group (60.0% versus 15.4%; Fisher’s exact test, *p* = 0.024).

Across the entire cohort, mean primary-tumor ADC was 941 ± 278× 10^−6^ mm^2^/s, with a median of 974.0 × 10^−6^ mm^2^/s and a range of 492–1532 × 10^−6^ mm^2^/s (Table 2). ADC measurements showed good intra- and interobserver reliability, with single-measure ICCs of 0.90 (95% CI, 0.88–0.95) and 0.81 (95% CI, 0.74–0.85), respectively.

### 3.2. Association Between ADC and Pathological T Stage

**Figure 2:**
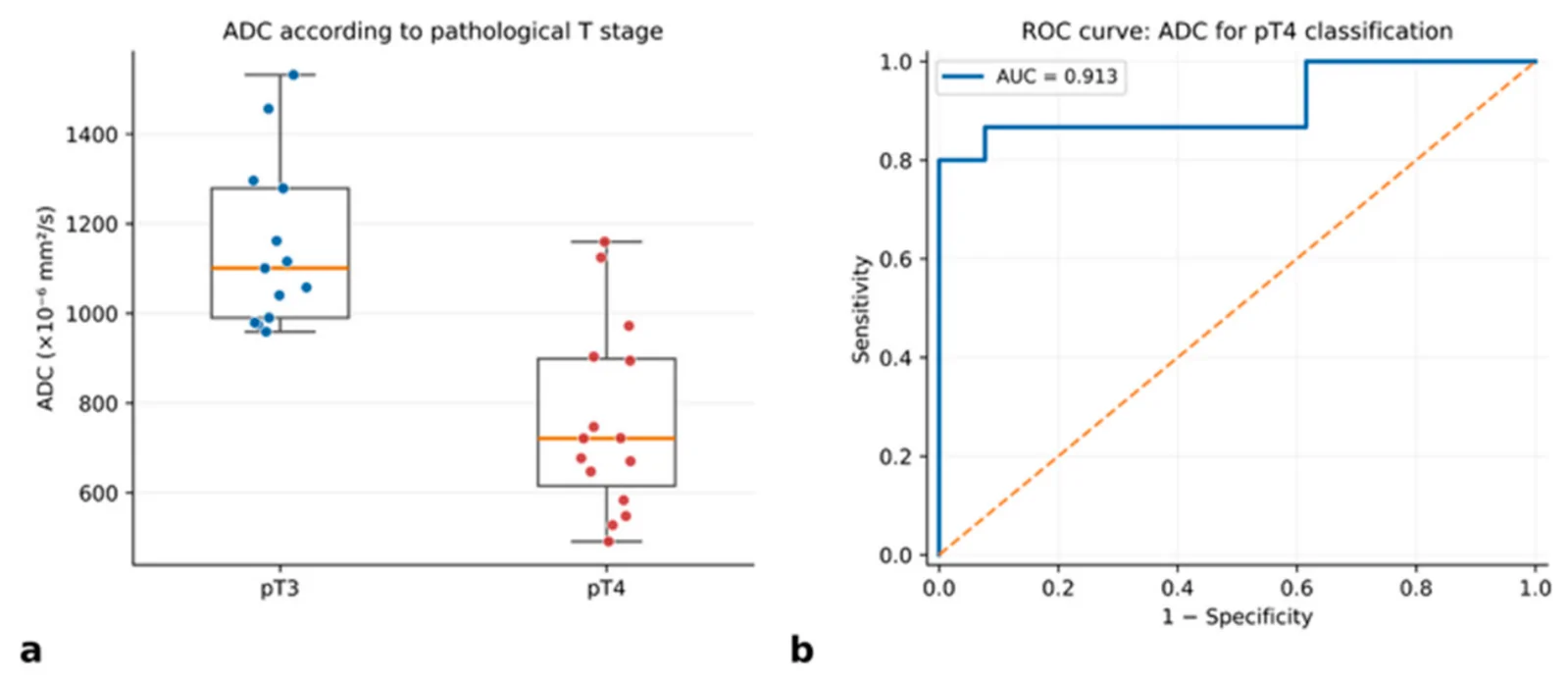
(**a**) Primary-tumor ADC distribution in pT3 and pT4 tumors, with individual patient values superimposed on boxplots. ADC values are expressed as ×10^−6^ mm^2^/s. (**b**) ROC curve for ADC discrimination between pT3 and pT4 disease. The dashed diagonal line represents the reference line for no discrimination. AUC, area under the ROC curve; ROC, receiver operating characteristic.

ADC values differed significantly between the pT3 and pT4 tumors (Figure 2a). Mean ADC was 1150 ± 188 × 10^−6^ mm^2^/s in the pT3 tumors and 760 ± 209 × 10^−6^ mm^2^/s in the pT4 tumors. Median ADC values were 1101 × 10^−6^ mm^2^/s and 722 × 10^−6^ mm^2^/s for the pT3 and pT4 tumors, respectively.

The Mann–Whitney U test showed significantly higher ADC values in pT3 than in pT4 tumors (U = 178; *p* < 0.001). The Hodges–Lehmann estimator indicated a median between-group difference of 393 × 10^−6^ mm^2^/s, calculated as pT3 minus pT4. The ADC distribution was shifted toward higher values in pT3 tumors, although partial overlap between the two groups was observed.

### 3.3. Discriminative Performance of ADC for pT4 Disease

ROC analysis showed that ADC discriminated between pT3 and pT4 tumors with an AUC of 0.913 (95% CI, 0.769–1.000) (Figure 2b). The exploratory threshold derived using the Youden index was 904 × 10^−6^ mm^2^/s. Using ADC ≤ 904 × 10^−6^ mm^2^/s to identify pT4 disease, sensitivity was 80.0% and specificity was 100%.

Univariable logistic regression demonstrated a significant inverse association between ADC and pT4 status (β = −1.081; OR per 100 × 10^−6^ mm^2^/s increase = 0.339; 95% CI, 0.148–0.777; *p* = 0.011), indicating decreasing odds of pT4 disease with increasing ADC values.

These values represented apparent in-sample performance because the threshold was selected and evaluated in the same cohort. During LOOCV, the threshold was 904 × 10^−6^ mm^2^/s in 26 out of 28 iterations and ranged from 894.67 to 972.33 × 10^−6^ mm^2^/s. The cross-validated ADC categorization correctly identified 11 out of 15 pT4 tumors and 12 out of 13 pT3 tumors, corresponding to a sensitivity of 73.3%, specificity of 92.3%, and overall accuracy of 82.1%.

### 3.4. MRI-Based Morphological T Classification and Exploratory Combined MRI–ADC Classification

Before consensus, the two radiologists agreed on the MRI-based morphological T classification in 23 out of 28 cases (82.1%) and disagreed in 5 cases (17.9%). Initial interobserver agreement was moderate, with a Cohen’s κ of 0.500 (95% bootstrap CI, 0.054–0.837). All disagreements were resolved by consensus. This agreement analysis reflects reader reproducibility and should not be interpreted as a measure of diagnostic accuracy, which was evaluated separately using the final consensus classification.

After completion of the blinded consensus assessment, the retrospective consensus MRI classification was concordant with the T stage reported in the original preoperative MRI report in all 28 patients. Neither retrospective reader had authored the original reports, which were accessed only after the consensus classification had been finalized. Initial interobserver agreement and concordance with the original reports represent different comparisons: the former was calculated from the readers’ independent assessments before consensus, whereas the latter was evaluated using the final consensus classification.

Among the 19 tumors assigned to the T3 category by MRI-based morphological T classification, 13 were confirmed as pT3, whereas 6 (31.6%) were classified as pT4 on histopathological examination. All nine tumors assigned to the T4 category by MRI-based morphological T classification were confirmed as pT4. Thus, MRI-based morphological T classification correctly identified 9 out of 15 pT4 tumors, while six were understaged as T3; none of the 13 pT3 tumors was overstaged as T4.

**Figure 3:**
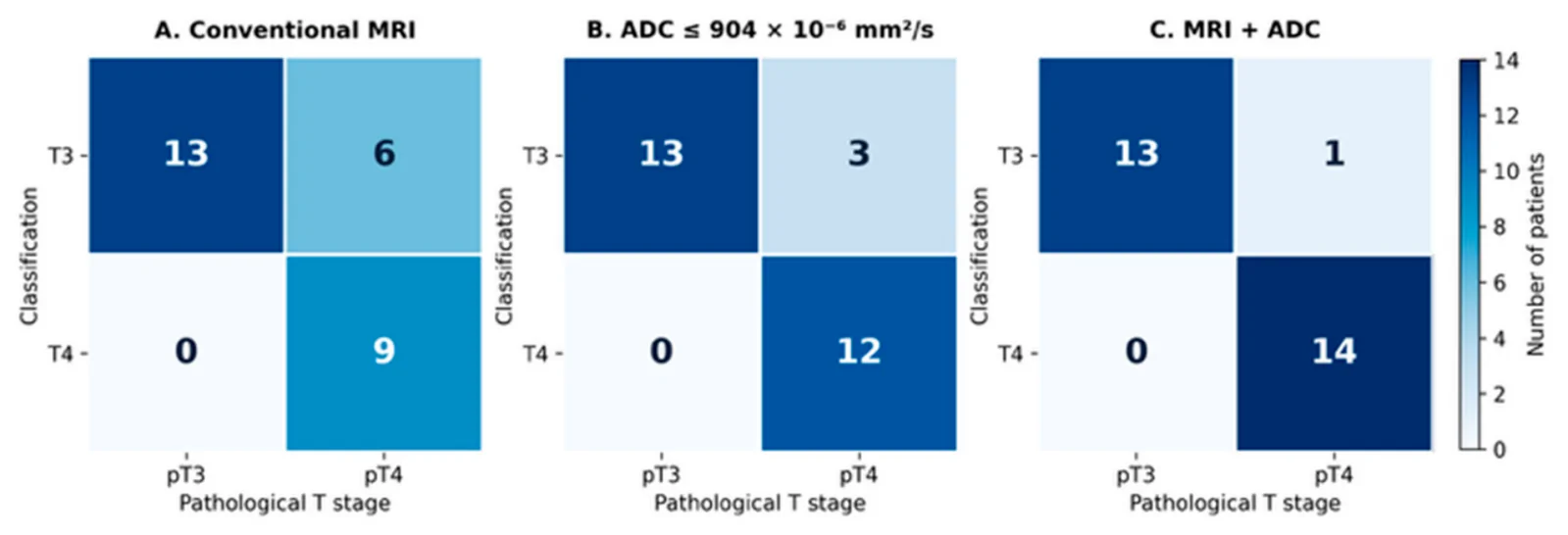
Confusion matrices for MRI-based morphological T classification, ADC thresholding (≤904 × 10^−6^ mm^2^/s), and the combined MRI–ADC approach for distinguishing pT3 from pT4 tumors. Columns indicate pathological stage and rows imaging-based classification.

ADC-based classification using the exploratory threshold of ≤904 × 10^−6^ mm^2^/s correctly classified 12 out of 15 pT4 tumors and all 13 pT3 tumors. The combined strategy, in which tumors were classified as pT4 when either MRI-based morphological T classification indicated T4 disease or ADC was ≤904 × 10^−6^ mm^2^/s, correctly classified 14 out of 15 pT4 tumors and all 13 pT3 tumors (Figure 3).

Apparent in-sample sensitivity was 60.0% for MRI-based morphological T classification, 80.0% for ADC threshold-based categorization, and 93.3% for the exploratory combined MRI–ADC rule. The corresponding specificity was 100% for all three approaches, while overall accuracy was 78.6%, 89.3%, and 96.4%, respectively (Table 3). Using the full-cohort threshold, the ADC criterion categorized five out of the six pT4 tumors understaged by MRI-based morphological T classification as pT4, whereas the MRI-based morphological assessment correctly identified two out of the three pT4 tumors missed by the ADC criterion alone. Cohen’s κ was 0.582 for MRI-based morphological T classification, 0.788 for ADC threshold-based categorization, and 0.929 for the combined rule. Discordance was unidirectional: six tumors were understaged by MRI-based morphological assessment, whereas no tumors were overstaged. The exact McNemar test yielded *p* = 0.031; however, because this result was based on only six discordant cases and no discordance in the opposite direction, it should be considered exploratory. The direction and magnitude of discordance are therefore more informative than the statistical significance of the test in this small cohort.

Because the ADC threshold and combined rule were developed and evaluated in the same cohort, these values represent apparent in-sample estimates and do not demonstrate superiority or validated incremental diagnostic benefit.

Under LOOCV, ADC threshold-based categorization correctly identified 11 out of 15 pT4 tumors and 12 out of 13 pT3 tumors, corresponding to a sensitivity of 73.3%, specificity of 92.3%, and overall accuracy of 82.1%. The cross-validated combined MRI–ADC rule correctly identified 14 out of 15 pT4 tumors and 12 out of 13 pT3 tumors, corresponding to a sensitivity of 93.3%, specificity of 92.3%, and overall accuracy of 92.9%.Thus, compared with the apparent in-sample estimates, LOOCV-based internal validation showed lower performance for ADC threshold-based categorization, while the combined rule showed the same sensitivity estimate but introduced one false-positive pT4 categorization among the 13 pT3 tumors. The corresponding exact 95% confidence intervals for sensitivity, specificity, and overall accuracy are reported in Table 3.

### 3.5. Association Between Primary-Tumor ADC and Pathological Nodal Status

**Figure 4:**
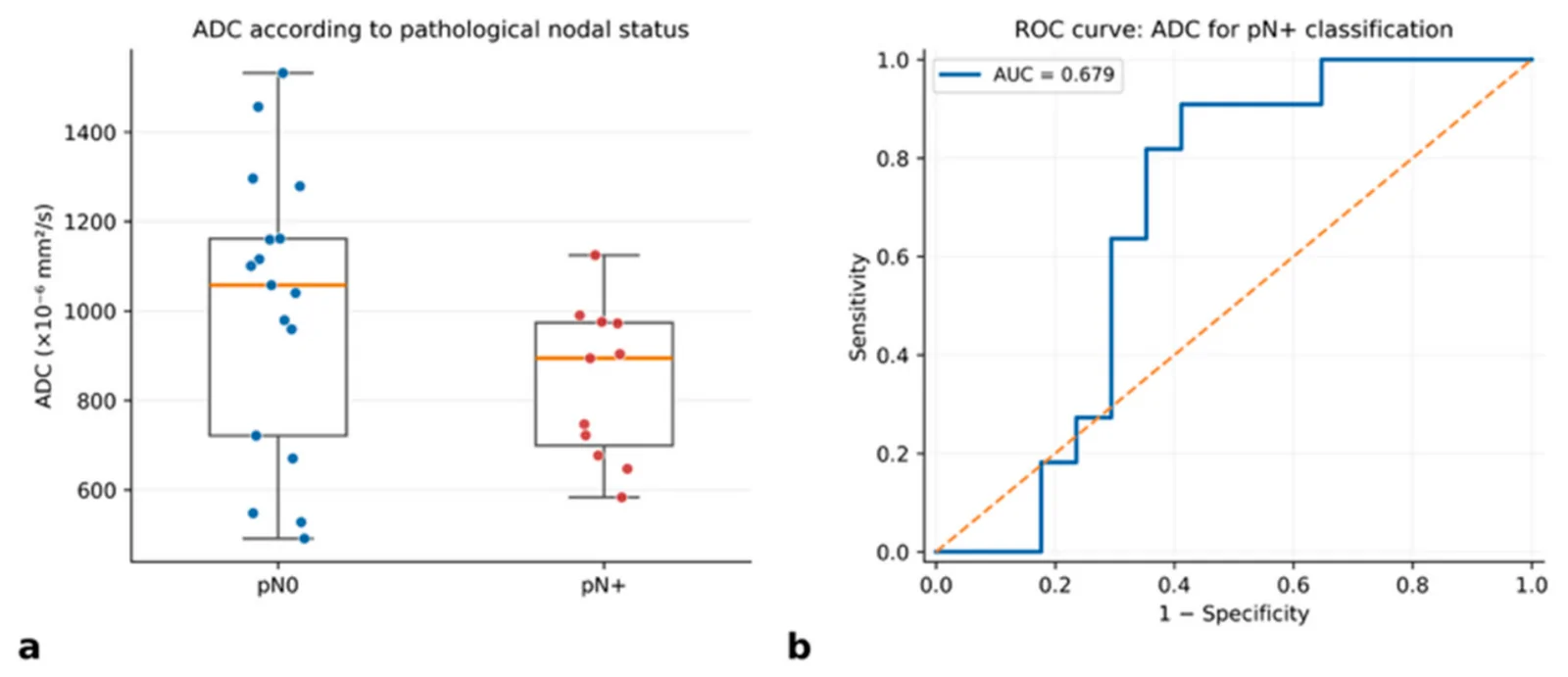
Primary-tumor ADC according to pathological nodal status: (**a**) ADC distribution in pN0 and pN+ tumors, with individual patient values superimposed on boxplots; ADC values are expressed as ×10^−6^ mm^2^/s; (**b**) ROC curve for identifying pN+ disease (AUC = 0.679). The blue and red dots represent individual patient ADC values in the pN0 and pN+ groups, respectively. The dashed diagonal line represents the reference line for no discrimination. AUC, area under the ROC curve; ROC, receiver operating characteristic.

Mean primary-tumor ADC was lower in patients with pN+ disease than in those with pN0 disease (840 ± 173 versus 1006 ± 317 × 10^−6^ mm^2^/s). The corresponding median values were 895 and 1058 × 10^−6^ mm^2^/s, respectively. However, the Mann–Whitney U test did not demonstrate a statistically significant between-group difference (U = 127; *p* = 0.121). The Hodges–Lehmann estimator indicated a location shift of 184 × 10^−6^ mm^2^/s, calculated as pN0 minus pN+ (Figure 4a).

ROC analysis yielded an AUC of 0.679, indicating limited discrimination between pN0 and pN+ disease (Figure 4b). Univariable logistic regression similarly showed no statistically significant association between primary-tumor ADC and pN+ status (β = −0.242; OR per 100 × 10^−6^ mm^2^/s increase = 0.785; 95% CI, 0.574–1.074; *p* = 0.130). No ADC threshold for pathological nodal status was derived.

## 4. Discussion

The principal finding of this study was the significant association between lower primary-tumor ADC and pT4 disease in a selected cohort of patients with moderately differentiated, locally advanced LSCC. ADC also showed good discrimination between pT3 and pT4 tumors, although the wide AUC confidence interval reflected substantial uncertainty related to the small sample size. Morphological MRI and ADC threshold-based categorization exhibited partly non-overlapping errors: using the full-cohort threshold, ADC categorized five out of the six pT4 tumors understaged by MRI as pT4, whereas MRI correctly identified two out of the three pT4 tumors missed by the ADC criterion. Under LOOCV, the combined rule identified 14 out of 15 pT4 tumors but introduced one false-positive pT4 categorization. This error pattern suggests potential complementarity between anatomical and diffusion-derived microstructural information but does not demonstrate superiority or validated incremental diagnostic benefit. The increased sensitivity of the combined rule should also be interpreted in light of its OR structure, which categorizes a tumor as pT4 when either component is positive and may therefore increase sensitivity at the expense of specificity. In the secondary exploratory analysis, no statistically significant association between primary-tumor ADC and pathological nodal status was detected. Accurate preoperative distinction between T3 and T4 disease remains clinically relevant because relatively subtle differences in cartilage penetration or extension beyond the laryngeal framework may substantially affect therapeutic planning and the feasibility of laryngeal-preservation strategies. Previous radiological–pathological correlation studies have documented limitations in the preoperative assessment of local tumor extent, including both understaging and overstaging [17]. In our cohort, 6 out of the 19 tumors classified as T3 by MRI-based morphological T classification (31.6%) were subsequently classified as pT4 on histopathological examination, whereas none of the 13 pT3 tumors was overstaged as T4 on MRI. The discordance was therefore unidirectional, with six cases of pathological upstaging from MRI-classified T3 to pT4 disease and no cases of MRI overstaging. The exact McNemar test yielded *p* = 0.031; however, this result was based on only six discordant cases and the complete absence of discordance in the opposite direction. It should therefore be considered exploratory and interpreted cautiously. It should be emphasized that staging in the present study was intentionally based on blinded MRI assessment, without access to laryngoscopic findings, PET/CT, other imaging examinations, or pathological results, and therefore represents MRI-based morphological staging rather than the complete multidisciplinary clinical staging process.

The six patients with MRI-understaged pT4 disease underwent surgical treatment, allowing definitive pathological staging. However, the retrospective MRI classifications generated for this study did not guide their clinical management. Treatment decisions had been made through the routine multidisciplinary process using the available clinical, endoscopic, and imaging information. Consequently, the present study cannot determine whether preoperative recognition of the definitive pT4 features would have changed the treatment selected for individual patients. Nevertheless, occult cartilage penetration or extralaryngeal extension at the T3–T4 boundary could potentially affect eligibility for laryngeal-preservation strategies or the extent of surgery. The clinical consequences of ADC-assisted staging should therefore be evaluated prospectively before its implementation in treatment planning.

Cartilage assessment represents a major source of uncertainty at the T3–T4 boundary. Morphological MRI is generally highly sensitive to cartilage abnormalities, but reactive inflammatory and perichondrial changes may mimic neoplastic infiltration and reduce specificity, whereas subtle cortical penetration or limited extralaryngeal extension may conversely remain difficult to recognize [18,19]. Refinement of MRI signal-intensity criteria can improve differentiation between neoplastic cartilage invasion and peritumoral inflammatory changes, but does not completely eliminate this diagnostic uncertainty [20]. Consequently, errors in morphological staging may occur in either direction, through overestimation or underestimation of local tumor extent [21]. The predominant pattern in our cohort was understaging: MRI-based morphological T classification correctly identified 9 out of 15 pT4 tumors, corresponding to a sensitivity of 60.0%, whereas specificity for pT4 disease was 100%. This finding does not contradict previous reports of high MRI sensitivity for cartilage invasion, because our endpoint was pathological T3–T4 classification, rather than cartilage invasion alone. Instead, it illustrates the broader difficulty of translating individual morphological findings—including cartilage penetration and extralaryngeal extension—into accurate preoperative T classification [22,23].

DWI may be relevant in this setting because it provides information different from the anatomical information obtained with morphological MRI. Previous histopathological correlation studies in laryngeal and hypopharyngeal squamous cell carcinoma have reported relationships between ADC and cellular density, extracellular space, nuclear characteristics, stromal composition, and tissue organization [24]. These established observations provide a biological rationale for the generally lower ADC values associated with increased cellularity and reduced extracellular space. However, cellularity, nuclear characteristics, and stromal composition were not quantitatively assessed in the present cohort. Consequently, these mechanisms should be regarded as biologically plausible explanations derived from previous literature rather than as histopathologically demonstrated mechanisms underlying the ADC differences observed in our patients.

In the present cohort, mean ADC was 760 ± 209 × 10^−6^ mm^2^/s in pT4 tumors and 1150 ± 188 × 10^−6^ mm^2^/s in pT3 tumors. This association suggests that pT4 status may be accompanied by measurable differences in tumor microstructure, but it does not establish a causal relationship between increasing anatomical T stage and decreasing ADC. Moreover, ADC should not be interpreted as a direct surrogate for T stage, cartilage penetration, or extralaryngeal extension. Pathological T classification describes anatomical tumor extent, whereas ADC reflects the tissue microenvironment influencing water diffusion.

The magnitude of the ADC values observed in our cohort is also biologically plausible within the existing head and neck diffusion literature. Malignant head and neck lesions consistently show lower mean ADC values than benign lesions, with pooled values around 1.0 × 10^−3^ mm^2^/s reported across heterogeneous malignant tumors [25]. Larynx-specific studies have similarly demonstrated restricted diffusion in carcinoma, compared with precursor or non-malignant lesions [26]. The overall mean ADC in our LSCC cohort (0.941 × 10^−3^ mm^2^/s) therefore lies within the expected range for malignant head and neck tissue. More importantly for the present study, pT4 tumors showed substantially lower values than the pT3 tumors (0.760 vs. 1.150 × 10^−3^ mm^2^/s). Previous diffusion studies have predominantly addressed lesion characterization, prognosis, or treatment response rather than pathological discrimination between T3 and T4 disease [27]. Our findings therefore address a different question: whether tumor microstructural information may contribute to identifying pathological local extension that is incompletely characterized by morphological imaging.

This distinction is important because the clinical significance of pretreatment ADC in HNSCC appears to depend strongly on the endpoint investigated. Its incremental value has been inconsistent when evaluated as a prognostic biomarker beyond established clinical variables [28]. The present findings should therefore not be interpreted as evidence for a general prognostic role of ADC. Rather, they suggest a potential application of ADC as an adjunct to morphological assessment of local tumor extent. This interpretation is also consistent with the absence of a significant association between primary-tumor ADC and pathological nodal status in our cohort, indicating that the information captured by ADC may not translate uniformly across different dimensions of tumor behavior.

ADC demonstrated good discrimination between the pT3 and pT4 tumors, with an AUC of 0.913 (95% CI, 0.769–1.000). The wide confidence interval, however, reflects the limited precision associated with the small sample size. Using the complete cohort, the exploratory Youden-derived threshold of ≤904 × 10^−6^ mm^2^/s yielded an apparent sensitivity of 80.0% and specificity of 100% for identifying pT4 disease. Under LOOCV, ADC threshold-based categorization showed a sensitivity of 73.3%, specificity of 92.3%, and overall accuracy of 82.1%, indicating lower performance than the apparent in-sample estimates. The threshold was relatively consistent within the present cohort, remaining at 904 × 10^−6^ mm^2^/s in 26 out of 28 LOOCV iterations and ranging from 895 to 972 × 10^−6^ mm^2^/s. However, this within-cohort consistency does not demonstrate robustness or generalizability, and the cutoff requires validation in an independent population. Absolute ADC values, however, are influenced by scanner characteristics, b-values, diffusion time, acquisition technique, ROI methodology, and post-processing procedures [29,30]. ADC measurements obtained using different acquisition strategies cannot therefore be assumed to be directly interchangeable. Accordingly, the threshold of ≤904 × 10^−6^ mm^2^/s should be regarded as a protocol- and cohort-specific, hypothesis-generating value rather than a clinically validated cutoff.

A clinically relevant descriptive observation was the partly non-overlapping pattern of errors between morphological MRI and ADC threshold-based categorization. Using the full-cohort threshold, the ADC criterion categorized five out of the six pT4 tumors understaged by MRI-based morphological T classification as pT4, whereas the MRI-based morphological assessment correctly identified two out of the three pT4 tumors missed by the ADC criterion alone. This pattern raises the hypothesis that morphological and diffusion-derived information may be non-redundant: MRI primarily characterizes anatomical tumor extension, whereas ADC provides quantitative information related to tumor microstructure. Under LOOCV, the combined MRI–ADC rule correctly identified 14 out of 15 pT4 tumors and 12 out of 13 pT3 tumors, corresponding to a sensitivity of 93.3%, specificity of 92.3%, and overall accuracy of 92.9%. Thus, LOOCV-based internal validation showed the same sensitivity estimate as the apparent combined analysis but introduced one false-positive pT4 categorization. These findings suggest potential complementarity but do not establish superiority or validated incremental diagnostic benefit. ADC should therefore be considered a potential adjunct for further investigation rather than a substitute for morphological MRI or an independently validated staging criterion. Independent external validation remains necessary.

**Figure 5:**
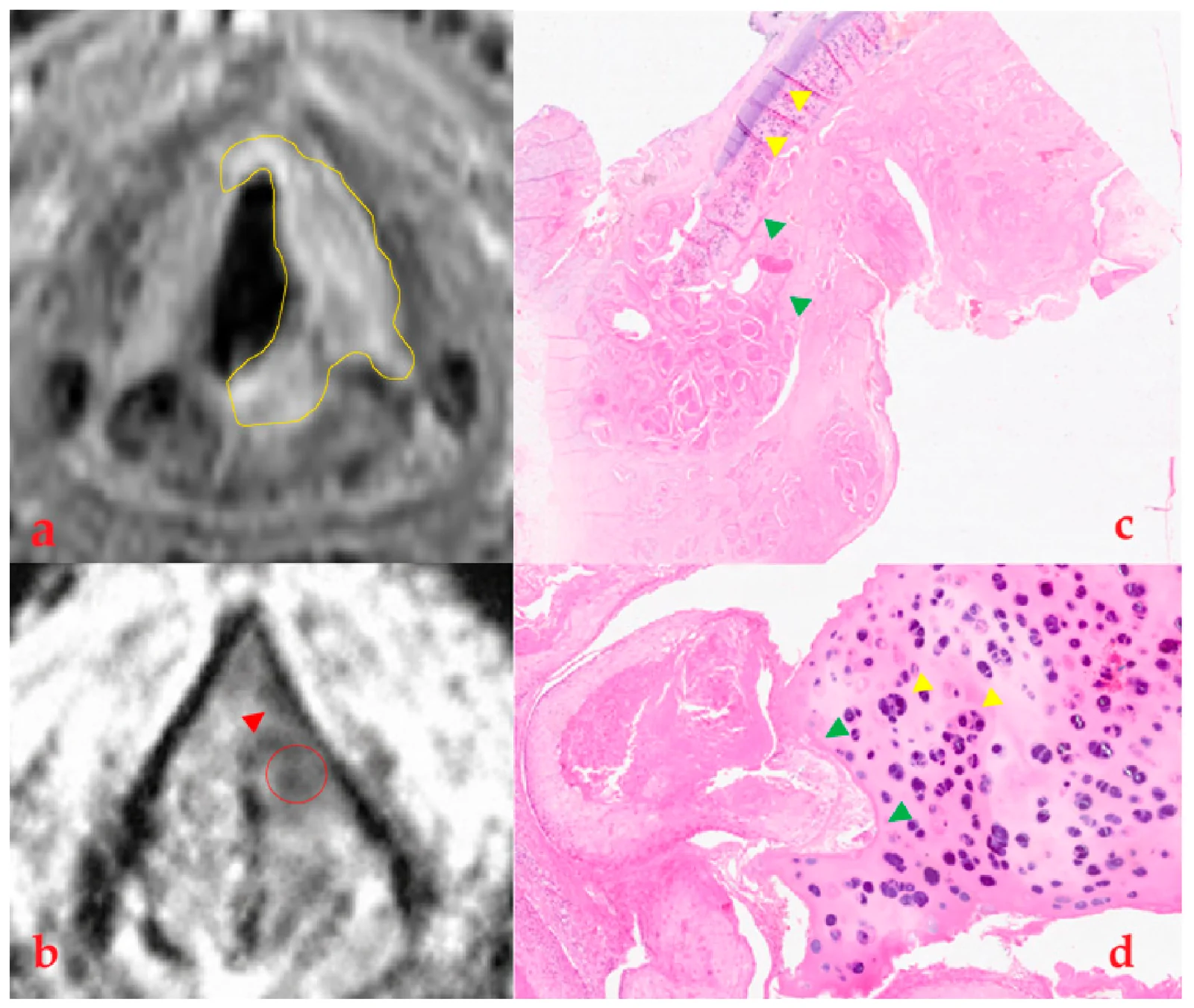
Radiological–pathological correlation in an MRI-understaged pT4a laryngeal squamous cell carcinoma. (**a**) Axial post-contrast fat-suppressed T1-weighted image showing the left glottic tumor, with the visible tumor boundary delineated by the yellow contour. The lesion was radiologically classified as T3N0, without definite evidence of full-thickness thyroid cartilage invasion or extralaryngeal extension. (**b**) Corresponding grayscale ADC map. The red arrowhead indicates the tumor region showing reduced diffusivity, while the red circle marks one of the three intratumoral ROIs used for ADC measurement. The other two ROIs were positioned on separate axial sections and therefore cannot be displayed in this single image. Tumor ADCmean, calculated by averaging the three ROI measurements, was 671 × 10^−6^ mm^2^/s. (**c**) Low-power hematoxylin and eosin-stained section (original magnification, ×2) showing the thyroid cartilage as a basophilic chondroid band (yellow arrowheads) and tumor extension through the cartilage toward the prelaryngeal soft tissues (green arrowheads). (**d**) Higher-magnification hematoxylin and eosin-stained section (original magnification, ×4) showing cartilaginous matrix with chondrocytes (yellow arrowheads) and the tumor–cartilage interface with infiltrating tissue (green arrowheads). Definitive pathological stage was pT4a pN0. Note. The histological fields shown are representative and were selected for illustrative radiological–pathological correlation; pathological staging was based on examination of the surgical specimen and was not limited to the displayed areas.

The radiological–pathological case presented in Figure 5 provides an illustrative example of the potential complementarity between morphological MRI and quantitative diffusion imaging. Morphological MRI demonstrated a heterogeneous, exophytic left glottic tumor with involvement of the anterior paraglottic space and focal subglottic extension. However, no definite thyroid cartilage invasion or extralaryngeal extension was identified, resulting in an MRI classification of T3N0. Tumor ADCmean was 671 × 10^−6^ mm^2^/s, below the internally derived exploratory cutoff of 904 × 10^−6^ mm^2^/s.

Histopathological examination following subtotal laryngectomy demonstrated moderately differentiated squamous cell carcinoma involving the glottic and subglottic regions, with focal necrosis, desmoplastic reaction, and moderate intra- and peritumoral inflammatory infiltration. In contrast to the morphological MRI assessment, the tumor infiltrated the full thickness of the thyroid cartilage and extended into the prelaryngeal soft tissues, where it was focally in contact with the India ink applied before tissue sampling (“ink on tumor”). All ten examined lymph nodes were negative for metastatic disease, resulting in a definitive pathological stage of pT4a pN0.

This individual case should not be interpreted as evidence that low ADC directly identifies cartilage invasion. Rather, it illustrates the hypothesis generated by the present findings: diffusion-derived microstructural information may identify tumors at risk of more advanced local extension, when the anatomical feature determining pT4 classification is not unequivocally recognized on morphological MRI [31,32].

Primary-tumor ADC was not significantly associated with pathological nodal involvement. Although pN+ tumors showed lower ADC values than pN0 tumors, the between-group difference was not statistically significant (*p* = 0.121); discrimination was limited (AUC = 0.679); and univariable logistic regression similarly showed no significant association with pN+ disease (OR = 0.785 per 100 × 10^−6^ mm^2^/s increase; 95% CI, 0.574–1.074; *p* = 0.130). Lymphatic metastatic potential reflects multiple biological processes, including lymphovascular invasion, angiogenesis, molecular alterations, and tumor–stroma interactions, which are unlikely to be captured by the mean ADC of the primary lesion alone. Moreover, ADC assessment performed directly within cervical lymph nodes addresses a different diagnostic question from the use of primary-tumor ADC as a surrogate for metastatic potential. The negative nodal findings therefore further support a cautious interpretation of ADC as a context-dependent imaging biomarker, rather than a general indicator of tumor aggressiveness. Given the small numbers of pN0 and pN+ patients, this secondary analysis was underpowered. Accordingly, the absence of statistical significance should not be interpreted as evidence that no association exists between primary-tumor ADC and pathological nodal involvement.

## 5. Limitations

This study has several limitations. Its retrospective, single-center design and small sample size limit statistical precision and generalizability. In particular, the limited number of pT4 events may have produced instability and overfitting in the univariable logistic-regression estimate, which should therefore be considered exploratory. Furthermore, the cohort was deliberately restricted to surgically treated patients with G2 pT3–pT4 LSCC. Although this restriction reduced heterogeneity related to histological type and grade, it resulted in a selected disease spectrum and prevents extrapolation to patients with early-stage tumors or G1 and G3 carcinomas. No direct quantitative histopathological correlation between ADC and tumor cellularity, nuclear characteristics, extracellular space, or stromal composition was performed in this cohort. Therefore, the biological mechanisms proposed to explain the lower ADC values observed in pT4 tumors were inferred from previous literature and were not directly demonstrated in the present study.

ADC assessment was based on three manually positioned ROIs rather than whole-tumor volumetric segmentation and may therefore incompletely capture spatial intratumoral heterogeneity. Nevertheless, the ADC measurements showed good intra- and interobserver reliability in the present cohort. ADC values may also be influenced by scanner characteristics, acquisition parameters, b-values, spatial resolution, image quality, and post-processing methodology, limiting their direct transferability across institutions.

Finally, leave-one-out cross-validation was performed to provide internal validation of the ADC threshold and exploratory combined MRI–ADC rule. Cross-validated performance was lower than the apparent in-sample performance, particularly for ADC threshold-based categorization, indicating some degree of optimism in the original estimates. Although the combined rule retained a sensitivity of 93.3%, its cross-validated specificity decreased from 100% to 92.3%. The Clopper–Pearson intervals for LOOCV estimates are descriptive and do not account for dependence among cross-validated predictions. However, internal validation within the same small, selected, single-center cohort cannot establish generalizability or transportability to other scanners, imaging protocols, institutions, or patient populations. The ADC threshold and combined MRI–ADC rule should therefore remain hypothesis-generating. Larger prospective multicenter studies using standardized DWI protocols, whole-tumor quantitative assessment, and independent external validation are required before ADC-based information can be integrated into clinical staging strategies.

## 6. Conclusions

In this selected cohort of patients with G2 pT3–pT4 LSCC, primary-tumor ADC was significantly lower in the pT4 than in the pT3 tumors and showed good discrimination between the two pathological T categories. MRI-based morphological T classification assessment understaged six out of 15 pT4 tumors. The partly non-overlapping errors of morphological MRI and ADC threshold-based categorization suggested potential complementary information. Under LOOCV, the exploratory combined MRI–ADC rule correctly identified 14 out of 15 pT4 tumors and 12 out of 13 pT3 tumors. However, these internally cross-validated estimates do not establish superiority or incremental diagnostic benefit. Primary-tumor ADC was not significantly associated with pathological nodal status. These findings support the further investigation of ADC as a complementary quantitative marker for local T staging, particularly in tumors at risk of morphological understaging. The proposed ADC threshold and combined MRI–ADC rule remain hypothesis-generating and require independent external validation in larger cohorts before clinical implementation.

## Figures and Tables

**Table 1:** Demographic and clinicopathological characteristics according to pathological T stage.

Characteristic	Overall Cohort (n = 28)	pT3 (n = 13)	pT4 (n = 15)	*p*-Value
Age, years				0.380
Mean ± SD	63.5 ± 8.2	62.0 ± 8.6	64.8 ± 7.8	
Median (range)	63 (44–82)	63 (44–76)	64 (51–82)	
Sex				0.600
Male	24 (85.7%)	12 (92.3%)	12 (80.0%)	
Female	4 (14.3%)	1 (7.7%)	3 (20.0%)	
Primary tumor site				0.276
Glottic	16 (57.1%)	9 (69.2%)	7 (46.7%)	
Supraglottic	12 (42.9%)	4 (30.8%)	8 (53.3%)	
Pathological nodal status				0.024
pN0	17 (60.7%)	11 (84.6%)	6 (40.0%)	
pN+	11 (39.3%)	2 (15.4%)	9 (60.0%)	
MRI-to-surgery interval, days, median (range)	26.5 (2–79)	27.0 (2–59)	26.0 (2–79)	0.821

Note. Data are presented as n (%) unless otherwise indicated. Percentages in the pT3 and pT4 columns were calculated within each pathological T-stage group. All pT4 tumors were classified as pT4a; no pT4b tumors were included. Age was compared using the Mann–Whitney U test; sex, primary tumor site, and pathological nodal status were compared using Fisher’s exact test. SD, standard deviation; pN0, absence of pathological nodal involvement; pN+, presence of pathological nodal involvement. Age and MRI-to-surgery interval were compared using the Mann–Whitney U test.

**Table 2:** Primary-tumor ADC values according to pathological T stage.

Group	n	Mean ± SD	Median	Range
Overall cohort	28	941 ± 278	974.0	492–1532
pT3	13	1150 ± 188	1101	959–1532
pT4	15	760 ± 209	722	492–1160

Note. ADC values are expressed as ×10^−6^ mm^2^/s. SD, standard deviation.

**Table 3:** Apparent and leave-one-out cross-validated diagnostic performance of MRI-based morphological T classification, ADC threshold-based categorization, and the exploratory combined MRI–ADC rule for identifying pT4 disease.

Imaging Method	Accuracy, % (95% CI)	Sensitivity,% (95% CI)	Specificity,% (95% CI)	PPV,% (95% CI)	NPV, % (95% CI)	Cohen’s κ
MRI	78.6 (59.0–91.7)	60.0 (32.3–83.7)	100 (75.3–100)	100 (66.4–100)	68.4 (43.487.4)	0.582
ADC	89.3 (71.8–97.7)	80.0 (51.9–95.7)	100 (75.3–100)	100 (73.5–100)	81.3 (54.496.0)	0.788
ADC, LOOCV	82.1 (63.1–93.9)	73.3 (44.9–92.2)	92.3 (64.0–99.8)	91.7 (61.5–99.8)	75.0 (47.6–92.7)	0.646
MRI + ADC	96.4 (81.7–99.9)	93.3 (68.1–99.8)	100 (75.3–100)	100 (76.8–100)	92.9 (66.199.8)	0.929
MRI + ADC, LOOCV	92.9 (76.5–99.1)	93.3 (68.1–99.8)	92.3 (64.0–99.8)	93.3 (68.1–99.8)	92.3 (64.0–99.8)	0.856

Note. Values are reported as percentages with Clopper–Pearson 95% intervals, except for Cohen’s κ. For LOOCV estimates, these are descriptive binomial intervals and do not account for the dependence among predictions arising from overlapping training sets. Apparent ADC and MRI–ADC estimates were calculated using the full-cohort threshold of ≤904 × 10^−6^ mm^2^/s. For leave-one-out cross-validation (LOOCV), the ADC threshold was recalculated in each 27-patient training set and applied to the corresponding held-out patient. Performance of the MRI-based morphological T classification was not cross-validated because this classification was not statistically fitted to the study dataset. ADC, apparent diffusion coefficient; CI, confidence interval; LOOCV, leave-one-out cross-validation; MRI, magnetic resonance imaging; NPV, negative predictive value; PPV, positive predictive value.

## Data Availability

The data supporting the findings of this study are available from the corresponding authors upon reasonable request and subject to applicable institutional approval. The data are not publicly available because they contain patient-level clinical, imaging, and histopathological information and are therefore subject to patient confidentiality and data-protection restrictions.

## Supplementary Materials

The following supporting information can be downloaded at: https://www.mdpi.com/article/10.3390/jimaging12100485/s1, Supplementary File S1: Completed STARD 2015 checklist.

## Abbreviations

The following abbreviations are used in this manuscript:

ADC	Apparent diffusion coefficient
AJCC	American Joint Committee on Cancer
AUC	Area under the receiver operating characteristic curve
CI	Confidence interval
CT	Computed tomography
DISCO	Differential subsampling with Cartesian ordering
DWI	Diffusion-weighted imaging
HNSCC	Head and neck squamous cell carcinoma
ICC	Intraclass correlation coefficient
LSCC	Laryngeal squamous cell carcinoma
MRI	Magnetic resonance imaging
NPV	Negative predictive value
OPHL	Open partial horizontal laryngectomy
OR	Odds ratio
PET/CT	Positron emission tomography/computed tomography
PPV	Positive predictive value
PROPELLER	Periodically rotated overlapping parallel lines with enhanced reconstruction
ROC	Receiver operating characteristic
ROI	Region of interest
SCC	squamous cell carcinoma
SD	Standard deviation
STROBE	Strengthening the Reporting of Observational Studies in Epidemiology
TE	Echo time
TR	Repetition time
